# Editorial: Immune Responses to Persistent or Recurrent Antigens: Implications for Immunological Memory and Immunotherapy

**DOI:** 10.3389/fimmu.2021.643989

**Published:** 2021-03-09

**Authors:** Stefano Caserta, Alejandra Pera

**Affiliations:** ^1^Department of Biomedical Sciences, The University of Hull, Hull, United Kingdom; ^2^Immunology and Allergy Group (GC01), Maimonides Institute for Biomedical Research of Cordoba (IMIBIC), Córdoba, Spain; ^3^Department of Cell Biology, Physiology and Immunology, University of Córdoba, Córdoba, Spain

**Keywords:** immunological memory, persistent antigens, immunosenescence, immunotherapy, cancer, sepsis, HIV, COVID-19

Immunological memory [for a critical, wider analysis of this concept refer to (1) and (2)] is generally considered a hallmark of the adaptive immune response, which is essential for long-term protection against infection throughout life. From the perspective of adaptive immunity, clonally expanded antigen-specific lymphocytes (T and B cells) accumulate within the immunological memory repertoire to confer protection upon re-encounter with persistent and/or recurrent pathogens. Furthermore, memory cells often respond more rapidly and effectively following antigen (Ag) encounter than naïve precursors do. Recent increasing evidence suggests that immunological memory can also be a feature of innate immune cells (3). Innate immunological memory has been frequently described as a trained potentiation of anti-pathogen responses upon re-infection and is exquisitely coordinated by transient genetic and transcriptional changes (e.g., epigenetic reprogramming) that alter the functions of innate immune cells, such as macrophages, monocytes, dendritic cells, and NK cells (3). The articles in this collection mostly focus on adaptive memory/memory-like cell responses development during chronic/endemic Ag exposure with implications for aging, infection, cancer, and therapy (Figure 1).

**Figure 1:**
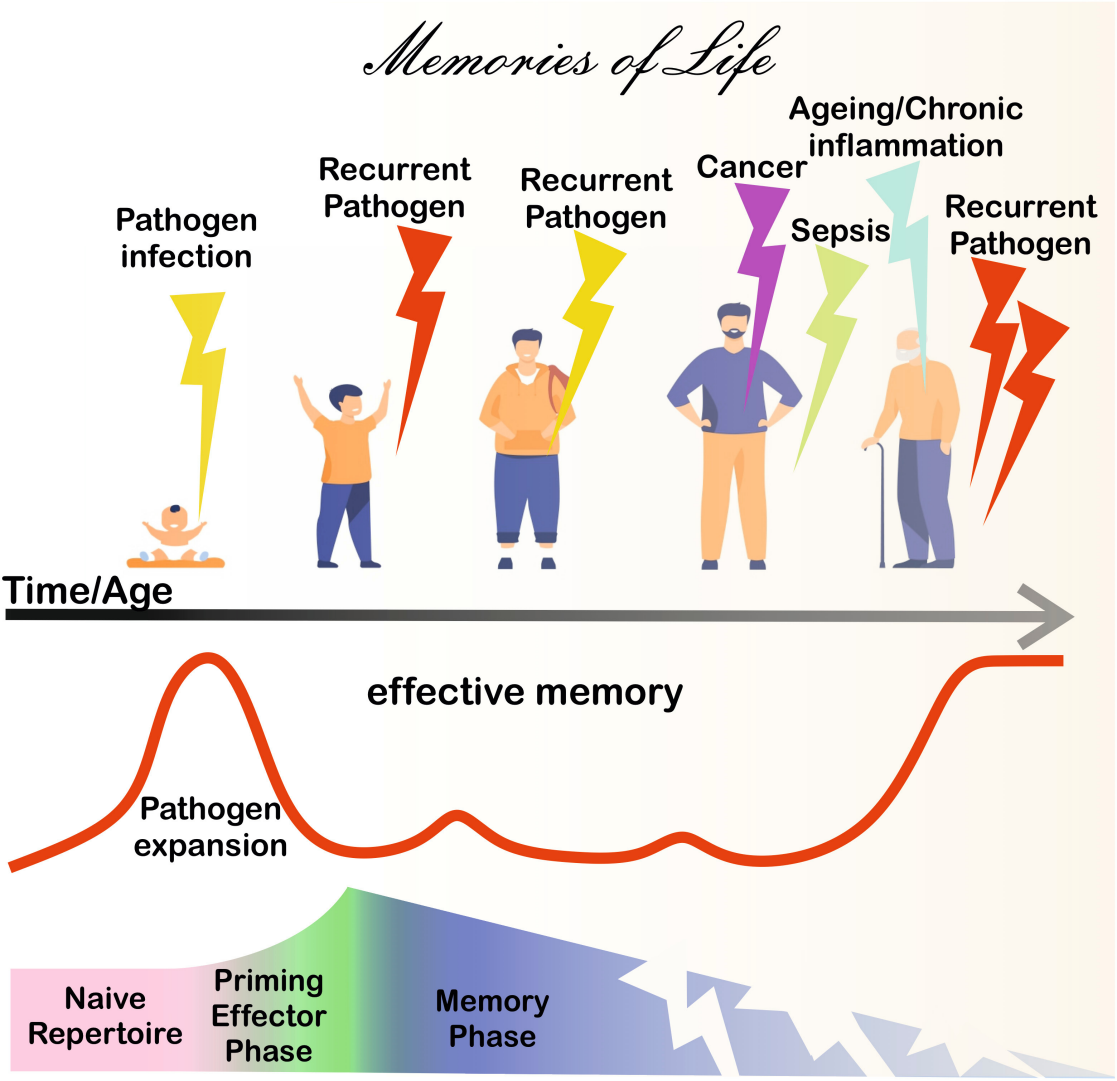
Immunological memory during life. After birth, pathogen-derived antigens (Ags) prime adaptive immunity cells from naive precursors (pink shadowed area), eventually leading to the formation of immunological memory (blue shadowed area), after the effector phase (green shadowed area). Memory cells protect in the long-term, however the memory repertoire is continually exposed to chronic Ag sources. For instance, chronic/recurrent inflammation and/or infection due to the presence of persistent/endemic pathogens or microbial Ag (bold red line) is likely to occur throughout life. Cancer disease can further expose adaptive cells to chronic tumor Ags. Severe infections, such as sepsis and COVID-19 are associated with immunoparalysis/exhaustion and/or loss of T cells. Ultimately, chronic/persistent stimulants can lead to a deterioration of immunity, leaving individuals more vulnerable to disease.

Under physiological conditions, throughout life, immunological memory responses undergo alterations implicated in (or perhaps even driving) the aging process. In their original review, Aiello et al. describe how the immune system changes during aging, relevant for vaccination efficacy, and other therapies aimed at reversing/delaying immune cell aging. For example, the authors review the application of dietary antioxidants and anti-inflammatory compounds (carotenoids, polyphenols, and polyunsaturated fatty acids); calory restriction and its drug mimetics (e.g., metformin); and micronutrients (such as zinc and vitamins). Further, they analyze strategies to reverse immune cell aging in vulnerable individuals, discussing the potential use of IL-7, as a growth factor to sustain naïve T cells, and checkpoint inhibitors as enhancers of T-cell responses, during aging. They explore the effects of microbiota (and dysbiosis) in immunotherapy linking this to the use of probiotics/prebiotics to limit inflammation, potentially useful to control inflammageing. The authors then discuss intracellular signaling pathways (p38/MAPK, sestrins/AMPK, and mTOR pathways) that could become targets of drug inhibitors, useful to limit/reverse (immune cell) aging, and new adjuvant formulations to boost vaccination efficacy in the elderly. Some of the changes associated with age are the result of adaption to stimuli, thus Aiello et al. stress the importance of targeted “rejuvenating” approaches. Indeed, memory responses to certain pathogens can deteriorate during aging, as seen for the age-associated decline of VZV-specific T cells. Contrarily, T-cell responses to other pathogens (most evidently, CMV) persist throughout life, reducing the diversity of the memory repertoire. Thus, both lifestyle and genetic factors influence immunosenescence and must be considered for treatment strategies.

The generation, persistence, and function of memory cells in humans during life may differ substantially from the responses seen in animal models where experimental variables are controlled in a reductionist approach, for example by deciding *a priori* the clonality of responder cells, the dose and modality of Ag exposure/administration, often without testing the impact of exposure to multiple infectious stimuli, as seen in real life conditions (4). Rather chronic/recurrent inflammation and/or infection due to the presence of persistent/endemic pathogens or microbial Ag are more likely to occur in humans, during life (1). Often (i) persistent or recurrent pathogens (e.g., virus, bacteria, and fungi); (ii) self-Ag derived from the body tissues; and (iii) cancer cells can lead to a deterioration of the immune response characterized by genetic/epigenetic alterations in immune cells driven by chronic or repeated exposure to Ags (Figure 1). In adaptive immunity, this constant stimulation by persistent Ags can lead to a disproportionate accumulation of Ag-experienced or memory-phenotype lymphocytes (memory inflation) (5). These phenomena can be associated with (i) a decreased diversity of Ag-receptor repertoires and (ii) alterations in signal transduction and cell differentiation processes, subsequently leading to dysfunctional responses, including exhaustion.

Relevant to Ag-receptor repertoires, in their original research article, Naumova et al. analyze the clonotype distribution within the circulating TCR-Vβ19^+^ CD8 T-cell pool (known to include influenza virus-specific T cells), during time. Under steady-state conditions (i.e., far from flu-like episodes), the blood T-cell repertoire comprises a dynamic component (potentially, tissue/depot resident cells entering the circulation) and many, low-frequency clone subpopulations which account for the larger fraction of the repertoire, stable over time. Such clonotype distribution may be reshaped by Ag-recall events in re-infected individuals, with repercussions on repertoire stability. Naumova et al. characterize the impact of Ag re-encounter on clonotype distribution in cultures derived from children, middle-age and older adults, at different time points. In terms of stability, the middle-age adults' repertoires are the most resilient, showing similar clonotype distributions between the recall and steady-state conditions. Hence, the generation of stable clonotypes in the repertoire relies on the maturation of the immune system over the years. In children, stable influenza-specific clonotypes are absent and recurrent (viral) Ag can drive loss of clonotypes that -the authors propose- would be replenished with new, best-fitting clonotypes. Toward adulthood, the T-cell repertoire would mature to reach an optimum of clonotype distribution and stability, capable to withstand Ag re-encounters, providing efficient protection against recurrent pathogens. However, such repertoire stability would erode in the face of repeated exposure to viral Ag, later in life. Especially in older individuals, the rate of clonotypes loss after recurrent infections would mark the deterioration of the repertoire and, hence, immunosenescence. This opens the interesting question as to how the skewness of the repertoire generated by real-life infections would impact on the responses, not only to the same, but also other pathogen-derived, cancer, and perhaps even self (cross-reactive?) Ags.

Covering cell differentiation processes in the context of chronic infections and persistent tumor Ags, Hope et al. present the challenges currently faced in the field to distinguish senescent and exhausted cells from memory counterparts. The authors discuss the surface markers (including inhibitory receptors: PD-1, TIM3, LAG-3, and others), the cytokines and the transcription factors (among many others: Blimp-1 vs. Bcl-6, Id2 vs. Id3, Eomes vs. T-bet, TOX, and Tcf-1) used to discriminate polyfunctional and/or long-term memory cells from the rest. They critically review memory-cell development/differentiation in patient infection studies and mouse models, from LCMV to SIV/HIV-1 and HCV, as well as *Trypanosoma cruzi, Toxoplasma gondii* and *Plasmodium* spp., and *Mycobacterium tuberculosis*, among others. They additionally draw important parallels with the case of chronic Ag responses during tumor disease, looking at melanoma, colorectal cancer, non-small cell lung carcinoma, and breast cancer. The changing horizons of T cell differentiation to chronic Ags can be decided by Ag load, time, and length of exposure, Ag removal and/or resolution of (including other) infection, cytokine milieu, concomitant inflammation, and anatomical cellular compartmentalization, with differences between CD8 and CD4 T cells. Further, CD4 T cells critically help memory CD8 T cell formation and B cell responses (especially in the case of T_FH_ responses), yet much more work is needed to characterize these during chronic infections and cancers. Therapeutically, in both cancer and infection, T-cell differentiation balances may be shifted with the application of antibodies directed to checkpoint receptors (anti-PD-1/PD-L1, anti-CTLA-4) to restore long-term responses, valid for both CD8 and CD4 T cells. In addition, vaccination with MHC-I/II-restricted Ag combined with specific adjuvants and/or concomitant depletion of regulatory T cells may prove beneficial to developing and maintaining responses to chronic tumor Ags.

Following on this thread, severe infectious conditions, such as sepsis, are also known to affect the metabolic profile and function of immune cells, somehow speeding up the exhaustion of memory-like cells. For example, patients affected by sepsis are more likely to have lifelong sequelae including the increased susceptibility to other subsequent infection (6), opening up the question of whether sepsis impacts on the memory immune repertoire with long-lasting impact. Relevantly, in their original research article, Niu et al. analyze inhibitory receptor expression in T cells during sepsis. They found that, in acute sepsis patients, an increased proportion of T cells express PD-1 *ex vivo* yet, later on (5 days from admission) these can further co-express LAG-3. This identifies a progression of T cell dysfunction/exhaustion during sepsis development. In recall responses to anti-CD3/CD28 and PMA/Ionomycin, sepsis-patient derived T cells that co-express both inhibitory receptors are less likely to secrete cytokines and proliferate, while showing increased trend to cell-death, relevant for sepsis immunoparalysis. Co-expression of PD-1 and LAG-3 on T cells is a relevant sepsis prognostic biomarker, as increased proportions of LAG-3^+^ PD-1^+^ T cells mark patients with more severe organ dysfunction, longer hospital stay, and diminished survival. Therapeutically, this study points at future avenues combining anti-PD-1 and anti-LAG-3 blockade, with specific timings to selectively prevent progression of exhaustion. Thus, future studies aimed at understanding the implications of T-cell differentiation and the wider impact that immunoparalysis can have on memory T cells in sepsis are critical to ameliorate therapy and manage patients, post-recovery.

Further on the theme of T-cell dysfunction in infection and cancer, the review by Vigano et al. discuss how T cell exhaustion is a common trait between HIV-1 infection and cancer. T-cell exhaustion is the consequence of Ag persistence, additionally instructed by immunoevasion mechanisms, particularly relevant in the tumor microenvironment. In both conditions, persistent activation induces TOX transcription factor which controls the transcriptional and epigenetic reprogramming of exhausted T cells. Another shared feature of T cell exhaustion in cancer and HIV-1 infection is the co-expression of several inhibitory immune checkpoint receptors (PD-1, CTLA-4, TIM-3, LAG-3, TIGIT, CD160, 2B4, and BTLA) by these cells. These changes translate into functional and survival defects that compromise T-cell effector functions and expansion capacity, while increasing susceptibility to apoptosis. Significantly, Vigano et al. cover differences affecting exhaustion in cancer and HIV-1 infection. For example, in HIV-1 infection, exhausted CD8 T cells express T-bet and Eomes which distinguish these cells from their progenitors, while in cancer the key transcription factors associated with exhaustion are Tcf-1 and STAT3. Similar to chronic viral infection, in cancer exhausted T cells have impaired functions. However, tumor infiltrating T cells are heterogeneous and can retain some degree of functionality that contribute to tumor control. This may explain the highly variable outcomes of immune checkpoint inhibitors therapy. Finally, the authors highlight the importance of discerning exhausted T cells from memory and activated cell phenotypes in order to design targeted immunotherapies. In this respect, inhibitory receptor expression is higher on exhausted rather than activated effector T cells, which express these receptors transiently. In addition, the transcriptional profiles of memory and exhausted T cells differ (*Rtp4, Foxp1, Ikzf2, Zeb2, Lass6, Tox*, and *Eomes*) and, in specific loci (e.g., *Pdcd1*), chronic Ag drives exhaustion-associated epigenetic imprinting that cannot reverse even after Ag decrease/removal. Understanding the processes involved in T-cell exhaustion during persistent stimulation by cancer or viral Ags is essential for the development of new immunotherapies.

With this in mind, the topic then develops into the therapeutic implications of immune memory for adoptive immunotherapy of cancer. In their review, Mondino and Manzo address the impact that pre-existing memories within the T-cell repertoire can have on the efficacy of cancer immunotherapy, in particular focusing on adoptive T-cell therapy (ACT), including chimeric Ag receptors (CAR) T cell therapy. During life, successive exposure to various Ags shapes the composition of memory (or Ag-experienced) immune repertoires in individuals. Bystander, cross-reactive, unrelated and/or suppressive memories instructed throughout the personal history of pathogen exposure will impact on future endogenous memory responses to cancer, as well as the efficacy of ACT. This would point at potential competition for environmental cues between endogenous pre-existing memory T cell clones and the transplanted cells. Factors to be considered in this respect span from clonal abundance and TCR affinity/avidity to availability of cytokines (particularly, common-γ chain cytokines) and nutrients (glucose, amino acids, and fatty acids). The authors discuss that pre-existing memory cells generated in response to previously encountered pathogens or cancer Ags in the initial stages of the disease could synergize with adoptively transferred T cells, in specific conditions. Yet, certain pre-existing memory cells could well-undermine the engraftment and dampen the efficacy of ACT cells, especially if they were to share characteristics of regulatory T cells. Authors propose that “good memories” will be cells with effector capabilities able to synergize with tumor-specific T cells provided by ACT. In this respect, proper activation of certain viral-specific memory T cells, could synergize with ACT. In contrast, “bad memories” would impair the development of new memories. Thus, repetitive encounters with the Ag could generate both good and bad memories, with opposite effects on the efficacy of ACT therapy. A thorough characterization of the host immunocompetence might help improving the efficacy of T-cell products, therefore increasing the probabilities of a successful therapeutic outcome.

The recent example of SARS-Cov-2 infection that has become endemic in the world is posing several interesting questions around memory cell persistence and function, in affected individuals. Although multiple vaccines may soon become widely available to hopefully protect against COVID-19, infection with SARS-Cov-2 is emerging as a new variable, drastically impacting on immune cells (7), potentially with long-term consequences for immunity. In their article, Diao et al. clearly show that immune cells, and in particular T cells are vastly reduced in total numbers in progressive COVID-19 disease stages. Such T-cell reduction is reminiscent of that happening in HIV^+^ individuals, but importantly further extends to CD8 T cells. This general loss of T cells would also affect individuals with mild infection symptoms and/or not requiring hospitalization. Interestingly, in COVID-19 patients, T-cell numbers inversely correlate with levels of inflammatory cytokines, IFN-γ, IL-6, and TNF-α, often described during cytokine storm reactions seen in sepsis and other systemic inflammatory diseases. The authors also show that, in the most severe forms of COVID-19, T cells would acquire an exhaustion phenotype. It remains unclear whether recruitment of T cells in other anatomical compartments (e.g., the lungs?) may explain loss of T cells during COVID-19. Nonetheless, drastic changes such as those described by Diao et al. would likely have an impact on the memory T cell compartment, potentially affecting, or even undermining, efficient responses to future pathogen encounters. Thus, it remains to be determined whether the phenomenon described by Diao et al. would help erase, change, and/or unbalance the historical record of immune memories within the repertoire of post-COVID-19 patients. Speculatively, it could be anticipated that such changes may play a role in long-COVID (8), and perhaps in future responses to cancer and recurrent pathogen Ags that post-COVID-19 patients will experience, later in life after recovery. Conversely, the weakening of the immune system so well-described in the elderly (Aiello et al.), and in patients suffering from cancer/HIV (Hope et al. and Vigano et al.) or sepsis (see Niu et al.), may predispose these individuals to severe COVID-19 disease, upon SARS-Cov-2 infection. It is still unclear whether individuals who experience non-severe or no symptoms after SARS-Cov-2 exposure might have cross-reactive memory T cells capable of mediating protection against this coronavirus (7). Recent evidence suggests the existence of cross-reactive Ags derived from other (more or less endemic) coronaviruses which may provide some degree of protection upon infection with SARS-Cov-2 (9). Again, fitting with this article collection, this would point at how recurrent exposure to endemic pathogens potentially helps create, shape, and even destroy our memories (Figure 1) with critical implications for health and disease, and treatment strategies.

## Author Contributions

SC and AP wrote the first manuscript draft and critically contributed to the final version of the manuscript. All authors contributed to the article and approved the submitted version.

## Conflict of Interest

The authors declare that the research was conducted in the absence of any commercial or financial relationships that could be construed as a potential conflict of interest.

## Abbreviations

Ag: antigen
ACT: adoptive T-cell therapy
CAR: chimeric antigen receptors.
